# BH3-only protein Bik is involved in both apoptosis induction and sensitivity to oxidative stress in multiple myeloma

**DOI:** 10.1038/sj.bjc.6605981

**Published:** 2010-11-09

**Authors:** L Bodet, E Ménoret, G Descamps, C Pellat-Deceunynck, R Bataille, S Le Gouill, P Moreau, M Amiot, P Gomez-Bougie

**Affiliations:** 1 Inserm, UMR892, Institut de Recherche Thérapeutique de l’Université de Nantes, Equipe 10 labellisée Ligue Nationale contre le Cancer 2008, 8 quai Moncousu, Nantes BP70721 F-44007, France; 2 CHU de Nantes, Service d’Hématologie Clinique, Nantes F-44000, France

**Keywords:** multiple myeloma, Bik, Bcl-2, TEF, apoptosis, oxidative stress

## Abstract

**Background:**

Although gene expression profile of multiple myeloma (MM) patients shows a wide range of Bik/Nbk expression, varying from absent to high, its regulation and function in myeloma cells is poorly understood. Thus, we addressed these questions in MM.

**Methods:**

Human myeloma cell lines (HMCLs) and primary purified myeloma cells were studied for Bcl-2 family protein expression by western blot and further correlation analysis was performed. Correlative study between Bik and thyrotroph embryonic factor (TEF) transcription factor expression was analysed by PCR. Stress oxidative response was analysed by flow cytometry.

**Results:**

A strong expression of Bik protein was found only in one out of three of HMCL and correlated to Bcl-2 expression (*P*=0.0006). We demonstrated that Bik could be regulated at the protein level by Bcl-2 and at the transcriptional level by TEF. Bik overexpression sensitises myeloma cells to oxidative stress whereas Bik silencing increases resistance to H_2_O_2_ oxidative stress. Furthermore, Bik ectopic expression disrupts Bim/Bcl-2 and Bim/Bcl-xL endogenous complexes triggering Bim release that could induce Bax and Bak activation.

**Conclusions:**

Ours results suggest that Bik has a role in both, apoptosis induction and sensitivity to oxidative stress in myeloma cells. Small BH3 mimetic molecules should be considered for further apoptosis-based therapy in myeloma cells expressing endogenous Bik/Bcl-2 complexes.

Multiple myeloma (MM) is a plasma cell malignancy presently incurable. It has a high degree of heterogeneity at presentation and a great variability with regard to the clinical outcome of patients in response to chemotherapy. Analyses of the global expression profile of patients have lead to the molecular classification of MM patients in different disease subtypes (Zhan *et al*, 2006). Recently, new therapy, such as proteasome inhibitors or immunomodulatory drugs have been introduced into MM treatment showing efficiency even in patients resistant to conventional chemotherapy (Ocio *et al*, 2008). However, despite these advances in MM treatment, all patients develop resistance to treatment that is often characterized by a reduced apoptosis rate. Thus, a better understanding of how a cell evades apoptosis is needed. Members of the Bcl-2 family are critical regulators of apoptosis and their ratio between pro-survival and pro-apoptotic members mainly determines the fate of a cell following an apoptotic stimulus. Pro-apoptotic BH3-only proteins are the most apical mediators of cell death and their pro-apoptotic activity is tightly regulated by diverse transcriptional and post-transcriptional mechanisms (Puthalakath and Strasser, 2002). BH3-only proteins can be subdivided in two groups, the sensitisers including Bmf, Bik/Nbk, Bad, Noxa and the activators Bim, Bid and Puma (Letai *et al*, 2002). Whereas activator BH3-only proteins can directly activate Bax or Bak, the role of sensitisers is to bind and occupy the antiapoptotic Bcl-2 proteins. As the gene expression profile of MM patients has demonstrated a wide range of Bik/Nbk expression, from absent to very high (Zhan *et al*, 2003), we addressed the regulation and the role of Bik in MM. Bik has been identified as a binding partner and antagonist of pro-survival Bcl-x_L_ and Bcl-2 and of two viral survival proteins, EBV-BHFR1 and adenovirus E1B-19 kDa (Boyd *et al*, 1995). Its BH3 domain is responsible of the cell death-promoting activity and of physical interactions with antiapoptotic molecules (Gillissen *et al*, 2003). Depending of the cellular context, Bik can be either strongly apoptotic when transfected in various cell lines (Han *et al*, 1996) or can sensitise tumour cells to apoptosis mediated by chemotherapeutic agents or by Fas (Daniel *et al*, 1999). Tight control of *Bik* gene expression at multiple steps is probably essential, given the fact that Bik harbours an already exposed BH3 domain (McDonnell *et al*, 1999). It has been proposed that induction of Bik by genotoxic stress is regulated at transcriptional level in a p53-dependent manner (Mathai *et al*, 2002). DNA methylation was also implicated in Bik transcriptional regulation (Sturm *et al*, 2006). More recently, it was shown that PAR bZIP proteins, a family of transcription factors, were able to regulate some BH3-only proteins (Ritchie *et al*, 2009). More specifically, one of these transcription factors, thyrotroph embryonic factor (TEF) directly controls Bik transcription. Indeed, chromatin immunoprecipitation assays indicate that TEF protein directly activates Bik promoter (Ritchie *et al*, 2009). In addition, the implication of Bik in the development of human breast and colorectal cancer was suggested because of the identification of a region deletion on chromosome 22q13, where *Bik* gene is located (Castells *et al*, 2000). Finally, post-transcriptional modifications were also implicated in Bik regulation. Thus, Bik activity can be regulated by phosphorylation which increases Bik pro-apoptotic capacity, probably by enhancing its binding affinity with antiapoptotic proteins Bcl-x_L_ and Bcl-2 (Li *et al*, 2003).

In this study, we first confirmed that Bik expression in human myeloma cells is very heterogeneous, varying from absent to very high and we next addressed the question of Bik regulation of expression and function in MM.

## Materials and methods

### Cells and culture conditions

KMS-11, KMM-1, KMS-12BM and KMS-12PE were kindly provided by Dr T Otsuki (Okayama, Japan), JJN-3 by Dr Van Riet (Belgium), JIM3 by Dr MacLennan (Birmingham, UK), Karpas 620 by Dr Karpas (Cambrigde, UK) and MM.1S by Dr Rosen (Chicago, IL, USA). LP-1, L363, NCI-H929 and OPM-2 human myeloma cell line (HMCL) were purchased from DSMZ (Braunschweig, Germany) and U266 from the ATCC (Rockville, MA, USA). The XG-1, XG-2, XG-5, XG-6, XG-7, NAN-1, NAN-3, NAN-6, SBN and BCN HMCL have been previously established in our laboratory from peripheral blood samples or pleural effusion of patients with MM and are cultured in the presence of 3 ng ml^−1^ of r–IL-6 (Novartis, Basel, Switzerland) (Bataille *et al*, 2006). Cell lines were maintained in RPMI-1640 medium supplemented with 5% fetal calf serum, 2 mM glutamine, antibiotics and 5 × 10^−5^M 2–*β*ME. Gradient density centrifugation using ficoll hypaque and purification by CD138-immunomagnetic beads were used to obtain purified malignant plasma cells from MM bone marrow specimens after written informed consent given at the University Hospital of Nantes. The purity of plasma cell population was always superior to 90% as assessed by morphology.

### Transient transfections and RNA interference assays

Transfection of KMM-1 was performed using Lipofectamin 2000 (Invitrogen Corp., Carlsbad, CA, USA). NCI-H929 and U266 HMCL were transfected using Amaxa Nucleofector (T solution, X01 programme and R solution, T01 programme, respectively). pcDNA3 Bik-HA and pRcCMV Bcl-2 were kindly provided by Dr G Chinnadurai, USA and by Dr F Vallette, France, respectively. Cells were transfected with 100 pmol siRNA using Lipofectamine 2000. All siRNA oligos used, namely, Bik (L-004388-00-0005), Bcl-2 (L-003307-00-0005), TEF (L-008769-00-0005), Bax (L-003308-01-0005) and Bak (L-009905-00-0005) were ON-TARGET plus siRNA pools of four oligos targeting four different mRNA regions at once and purchased from Dharmacon (Chicago, IL, USA).

### Antibodies (mAbs) and reagents

Anti-Bcl-2 (clone 124) mouse mAb was obtained from Dako (Roissy, CDG, France). Antibodies against caspase-3 (clone E-8) mouse mAb, Bik (N-19) goat polyclonal, caspase-9 (clone F-7) mouse mAb and Mcl-1 (S19) rabbit polyclonal were obtained from Santa Cruz Biotechnology (Tebu-Bio, Le Perray en Yvelines, France). Antibodies against PUMA rabbit polyclonal was from Calbiochem (Merck, Darmstadt, Germany). Anti-Actin (clone C4) and anti-Bim rabbit polyclonal antibodies were obtained from Chemicon (Temecula, CA USA). Anti-Noxa (clone 114C307.1) from Alexis (Coger, Paris, France).

### Apoptotic cell death

Cell death was assessed by Apo 2.7 (Beckman Coulter, Marseille, France) staining. Flow cytometry analysis was performed on a FACSCalibur using the Cell Quest software (Becton Dickinson, San Jose, CA, USA).

### Immunoblotting

Cells (5 × 10^6^) were resuspended in 150 *μ*l lysis buffer (10 mM Tris, pH 7.6, 150 mM NaCl, 5 mM EDTA and 1% Triton-X 100) containing 2 mM PMSF and 2 *μ*g ml^−1^ aprotinin. After 40 min on ice, lysates were cleared by centrifugation at 12 000 **g** for 30 min at 4 °C. Equal amounts of total protein were separated by SDS–PAGE, electrotransferred onto PVDF membranes and analysed following standard procedures. The signal was detected by ECL detection (Pierce, Rockford, IL, USA).

### Immunoprecipitation

Cells (25 × 10^6^) were lysed in 1% digitonin containing lysis buffer. Whole-cell lysates were obtained, pre-cleared with protein A-sepharose and then incubated overnight with 5 *μ*g of the specific antibody. Immunocomplexes were captured with either protein A-sepharose or protein G-agarose. Beads were pelleted, washed three times and boiled in SDS sample buffer. The presence of immune complexes was determined by western blotting analysis.

### RT–PCR and sequencing

Total RNA was prepared using Nucleospin RNA II (Macherey-Nagel Corporation, Düren, Germany). For complementary DNA synthesis, 2 *μ*g of the total RNA was reverse transcribed using the Moloney murine leukemia virus reverse transcriptase (Invitrogen Corp.,) and oligo-(dT)12-18 (Invitrogen). Amplification of *TEF*, *Bik* and *Actin* was performed using the following primers: *TEF* forward (5′-TGGTCCTGAAGAAGCTGATGG-3′) and reverse (5′-TCCAGGTCCATGTACTCCAG-3′), *Bik* forward (5′-GACCATGGAGGTTCTTGGCA-3′) and reverse (5′- AGGCTCACGTCCATCTCGTC-3′) and *Actin* forward (5′-ATCTGGCACCACACCTTCTACAATGAGCTGCG-3′) and reverse (5′-CGTCATACTCCTGCTTGCTGATCCACATCTGC-3′). P53 mutations were identified by direct sequencing of RT–PCR products. For p53, two overlapping amplifications, a 750 pb (codons 1–250) and a 819 pb (codons 187–370) products, were performed using the following primer pairs: ATGGAGGAGCCGCAGTCA and GGCCTCCGGTTCATGCCG (1–250), GGCCCCTCCTCAGCATCTT and TCCCCATCCTCCTCCCCA (187–370).

#### Detection of ROS generation

For ROS generation analysis, cells were exposed to 200 *μ*M H_2_O_2_ during 2 h and then incubated with 5 *μ*M dihydroethidine (DHE) (Molecular Probes, Eugene, OR, USA) at 37 °C for 15 min. After incubation, cells were washed twice with ice-cold phosphate-buffered saline. Oxidation of DHE to oxyethidium was then determined by flow cytometry.

## Results

### Bik expression appears to be correlated to Bcl-2 expression and lack of Bik expression is not related to lack of other BH3-only protein

**Figure 1:**
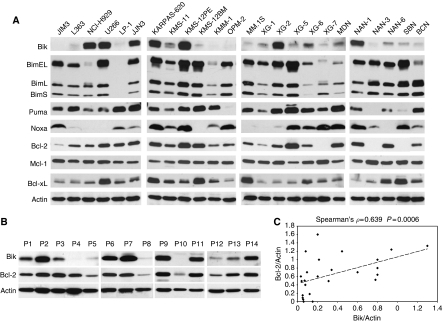
Bik expression is correlated with Bcl-2 expression. (**A**) Expression of BH3-only pro-apoptotic (Bik, Bim, Puma and Noxa) and antiapoptotic (Bcl-2, Mcl-1, Bcl-x_L_) proteins on HMCL. Equivalent amounts of cell lysates (70 *μ*g of protein) were subjected to immunodetection with the indicated antibodies. Protein loading was controlled by probing with an anti-actin antibody. (**B**) Expression of Bik and Bcl-2 proteins on purified primary myeloma cells. (**C**) Bik and Bcl-2 from HMCL were quantified using Image-J software, both proteins were normalised by actin.

We used a fairly large number of HMCL (*n*=24) to analyse Bik expression. We found that only 9 HMCL (37.5%) displayed a moderate to strong expression of Bik protein. In contrast, weak expression or complete absence of Bik protein was observed in the other HMCL (Figure 1A). We also analysed Bik expression in CD138+ primary myeloma cells. Among 14 purified samples, Bik protein levels were moderate to high in eight, and low to absent in six primary myeloma samples. We, and others, have demonstrated that pairs like Mcl-1/Bim and Bcl-2/Bim may mutually regulate their expression (Jorgensen *et al*, 2007; Toumi-Wuillème *et al*, 2007). In view of these findings, we were interested to establish whether the absence or weak Bik expression detected in 62.5% of HMCL was related to the expression of Bcl-2 or Bcl-x_L_. Western blotting and further quantification analysis showed that Bik expression was directly correlated to Bcl-2 protein expression in both HMCL (Spearman's *ρ*=0.639 *P*=0.0006) (Figure 1C) and primary myeloma cells (Spearman's *ρ*=0.815 *P*=0.0005). However, no significant correlation was found between Bik and Bcl-xL expression (Spearman's *ρ*=0.079 *P*=0.711). Apart from Bik, we analysed the expression of other BH3-only proteins, as loss of Bik was previously shown to coincide with lack of Bim, Noxa and Bad in renal cell carcinoma (Sturm *et al*, 2006). Most HMCL expressed readily detectable levels of all three Bim isoforms EL, L and S (76%). Bad was constitutive and homogeneously expressed by all HMCL analysed (not shown). Despite of low or negative Noxa expression detected in around half of HMCL, no direct correlation with Bik expression was observed (Figure 1A). Altogether, these results suggest that in myeloma cells, Bik expression is directly correlated to Bcl-2 levels whereas there is no relationship between Bik expression and the expression of any other BH3-only protein studied.

### Bcl-2/Bik complexes are involved in Bik protein stability

**Figure 2:**
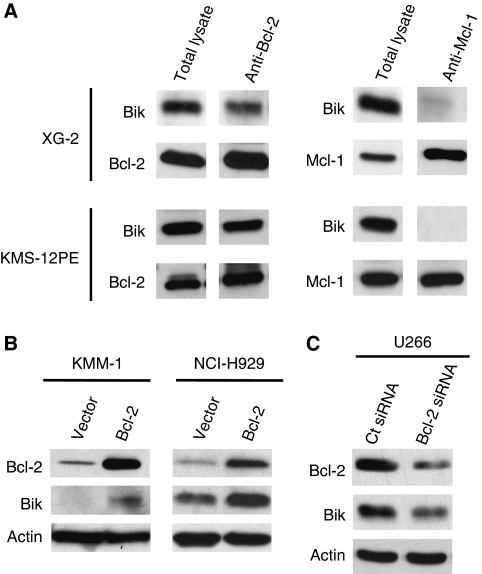
Bcl-2 expression stabilises Bik protein. (**A**) Bik and Bcl-2 form endogenous complexes in XG-2 and KMS-12PE HMCL. Lysates were subjected to immunoprecipitations with anti-Bcl-2 or anti-Mcl-1 antibodies and immunoblotting for the indicated proteins. (**B**) Ectopic expression of Bcl-2 stabilises Bik protein. Cells were transfected with pRcCMV-Bcl-2 or an empty vector. At 48 h after transfection, cells were harversted. Extracts were then immunoblotted for the indicated proteins. (**C**) Bcl-2 silencing downregulates Bik expression. U266 cells were transfected with control or Bcl-2 siRNA, protein expression was determined 24 h after transfection.

As BH3-only molecules are kept in check by the antiapoptotic Bcl-2 proteins, we assessed the presence of constitutive Bik complexes in XG-2 and KMS-12PE cells. Figure 2A shows that myeloma cells displayed constitutive complexes of Bik essentially with Bcl-2 rather than with Mcl-1. Taking into account the above results, either the direct correlation between Bik/Bcl-2 expression or the detection of endogenous Bik/Bcl-2 complexes, we assessed the possible regulation effect of Bcl-2 expression on Bik levels. Thus, overexpression of Bcl-2 in myeloma cells induced an accumulation of Bik (Figure 2B). On the contrary, Bik overexpression did not induce Bcl-2 accumulation (not shown). To confirm the stabilisation of Bik by Bcl-2 in MM, we showed that downregulation of Bcl-2 by siRNA in U266 cells induced a decrease of Bik expression (Figure 2C).

### mRNA Bik expression correlates with the expression of TEF mRNA independent of p53 status

**Figure 3:**
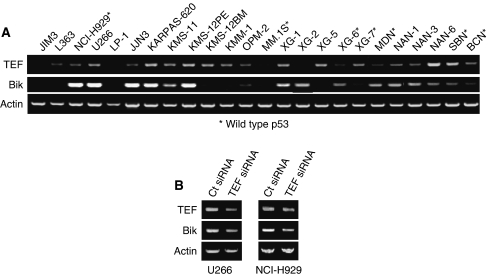
TEF transcription factor expression is correlated to Bik levels. (**A**)The mRNA levels of *TEF* and *Bik* of 24 HMCL were analysed by RT–PCR. *Actin* mRNA was used as an amplification control. Wild-type p53 HMCL are marked by ^*^. (**B**) U266 and NCI-H929 cell lines were transfected with non-target control or TEF-specific siRNA. The mRNA levels were analysed by RT–PCR 72 h after transfection.

Several transcription factors have been involved in the transcription of *Bik*. More particularly, the tumour suppressor p53 was implicated in the induction of Bik (Mathai *et al*, 2002). Therefore, we compared the status of p53 of HMCL (Supplemental data) with Bik expression showing no direct correlation between basal expression of Bik protein and the status of p53 (Figure 3A). More recently, it was demonstrated that TEF transcription factor directly binds the promoter of *Bik* and controls its transcription. Thus, we investigated the expression of *TEF* and *Bik* in our HMCL collection by RT–PCR. Of interest, as shown in Figure 3A, the analysis of this large collection of cell lines showed that *Bik* is expressed only in the presence of *TEF* mRNA, except for XG2 cell line, indicating that *Bik* gene could be transcriptionally activated by TEF in myeloma cells. To validate this hypothesis, the effect of *TEF* silencing on *Bik* expression was assessed (Figure 3B). In myeloma cells, the downregulation of *TEF* mRNA triggers an important *Bik* decrease, confirming the role of TEF in *Bik* transcription.

### Bik is implicated in oxidative stress response

**Figure 4:**
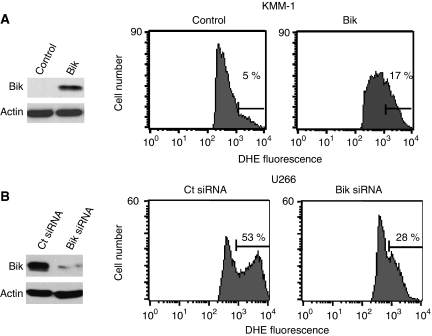
Bik contributes to the oxidative stress response. (**A**) Transient expression of Bik sensitises KMM-1 to oxidative stress. Cell line KMM-1 was transiently transfected with Bik complementary DNA or an empty vector (control), after 48 h, expression levels of Bik protein were determined by western blot. Transfected cells were treated with 200 *μ*M H_2_O_2_ and then ROS generation was measured by dihydroethidine (DHE) staining followed by fluorescence-activated cell sorting analysis. (**B**) Bik silencing promotes protection to oxidative stress. U266 were transfected with non-target control or Bik-specific siRNA, 24 h after transfection, lysates were obtained and analysed for Bik expression levels. Transfected cells were treated with 200 *μ*M H_2_O_2_ and stained as above. Results are representative of three independent experiments.

As it was demonstrated that TEF participates in oxidative stress responses via Bik expression (Ritchie *et al*, 2009), we assessed the role of Bik in ROS generation. We transfected KMM-1 myeloma cells with 0.5 *μ*g Bik complementary DNA leading to moderate Bik ectopic expression that sensitises KMM-1 cells to oxidative stress, as shown by the increase of oxyethidium formation (Figure 4A). In agreement with this result, the silencing of Bik rendered U266 cells resistant to oxidative stress, as indicated by the decrease of highly DHE-positive cells from 53 to 28% (Figure 4B). Altogether, these results show the implication of Bik in H_2_O_2_ oxidative stress responses in myeloma cells.

### Transient ectopic expression of Bik induced cell death

**Figure 5:**
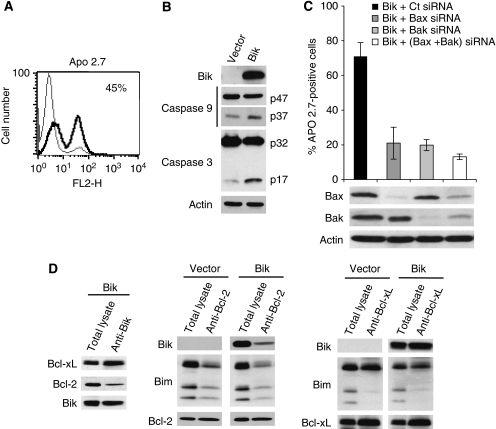
Bik overexpression induces cell death in myeloma cells. (**A**) KMM-1 cells were transfected either with empty vector (thin line) or Bik (thick line) complementary DNA, after 48 h cell death was measured by Apo2.7 staining. Results are representative of three independent experiments. (**B**) Cell lysates were analysed by western blotting analysis to assess caspase-9 and caspase-3 activation. (**C**) Cells were co-transfected with Bik cDNA in the presence of control, Bax, Bak or Bax and Bak siRNA. At 48 h after transfection, cells were analysed for Apo2.7 staining. Expression levels of Bax and Bak were determined by western blot. (**D**) KMM-1 cells were transfected either with empty vector or Bik cDNA, 24 h after transfection cells were harvested and cell lysates were obtained. Bik, Bcl-2 and Bcl-xL immunoprecipitates were analysed by western blot.

To address the effect of ectopic expression of Bik, myeloma cells were transiently transfected either with Bik or with an empty vector. Ectopic Bik expression induced cell death detected by Apo 2.7 staining and activation of both caspase-9 and caspase-3 (Figure 5A and B). To explore whether Bax or Bak was involved in the pro-apoptotic function of Bik, silencing of Bak and Bax were performed and resulted in a complete decrease of Bax and Bak expression (Figure 5C). Knockdown of either Bak or Bax correlated with a strong decrease of Bik induced apoptosis, 72 and 75% respectively. These results indicate that overexpression of Bik promotes cell apoptosis through a Bak /Bax-dependent mitochondrial pathway. To unravel the mechanism of Bik-induced cell death, we analysed its binding partners under overexpression in KMM1 cells by co-immunoprecipitation experiments. Although ectopic Bik expression led to the formation of both Bcl-x_L_/Bik and Bcl-2/Bik complexes, we observed a dissociation of Bcl-2/Bim and Bcl-x_L_/Bim complexes as shown in Figure 5D. Thus, these findings suggested that Bim is released from Bcl-xL and Bcl-2 and became available to exert its pro-apoptotic function.

## Discussion

The importance of BH3-only pro-apoptotic proteins in cancer therapy has become increasingly evident, and a wide variety of transcriptional and post-translational mechanisms that regulate their expression and activity has been reported (Puthalakath and Strasser, 2002). In this report, we found that Bik levels are very heterogeneous from absent to very high in both, cell lines and primary myeloma cells. We first looked for a relationship between Bik and other Bcl-2 family members. We provide evidence that there is no relationship between the absence of Bik and the presence of either Bim or Bad, as these two molecules are always expressed in MM cells with the exception of Bim in one cell line. Furthermore, while Noxa is absent in about 50% of HMCL, there is no direct correlation between the lack of expression of Noxa and Bik. The relationship of BH3-only proteins expression in myeloma cells appears very different to the observation reported in renal cell carcinoma, where loss of Bik coincides with lack of Bim, Noxa and Bad (Sturm *et al*, 2006). Interestingly, we found that Bik expression is directly correlated to Bcl-2 protein expression in both HMCL (*P*=0.0006) and primary myeloma cells (*P*=0.0005). In contrast, there is no significant correlation between Bik and Bcl-x_L_. We also demonstrated that viable MM Bik-positive cells exhibit endogenous abundant Bik/Bcl-2 complexes whereas no Mcl-1–Bik interaction was detected. We noticed that some cell lines expressing very high levels of Bik and Bcl-2 harbour t(11;14) as U266, KMS-12PE and Karpas 620. Interestingly, MM patients harbouring this translocation do not belong to the worst prognosis group (Avet-Loiseau *et al*, 2002). We, and others, have demonstrated that some Bcl-2 family protein pairs may mutually regulate their expression. Pairs like Mcl-1/Bim and Bcl-2/Bim support this proposal (Jorgensen *et al*, 2007; Toumi-Wuillème *et al*, 2007). In view of these findings, we overexpressed Bcl-2, which induced an important accumulation of Bik. Moreover, silencing of Bcl-2 triggers Bik downregulation, suggesting a direct effect of Bcl-2 on Bik protein expression. According to this, Mathai *et al* demonstrated in oral epithelial carcinoma cells that Bik expression was directly influenced by the expression of Bcl-2 in an inducible system. They suggested, using this model, that failure to observe Bik induction in the absence of Bcl-2 may result from Bik degradation (Mathai *et al*, 2002). Altogether, these results suggested that a very high expression of Bik could be only the reflection of an adaptation of the tumour to high Bcl-2 levels. Besides the stabilisation of Bik by Bcl-2 at the protein level, it was previously proposed that p53 indirectly influence Bik expression (Mathai *et al*, 2002). We demonstrated that there is no direct correlation between basal expression of Bik and the status of p53, however we cannot rule out that Bik expression may be induced once a genotoxic signal activates p53 in wild-type cell lines. More recently, Ritchie *et al* demonstrated a transcriptional regulation of *Bik* by TEF, a PARbZIP transcription factor, showing by both promoter-reporter and chromatin immmunoprecipitation assays that TEF protein activates directly *Bik* promoter (Ritchie *et al*, 2009). In agreement with this finding, we showed that *Bik* is expressed only in the presence of *TEF* mRNA. Consistently, we demonstrated that TEF silencing triggers a strong decrease of *Bik* mRNA, confirming that *Bik* is transcriptionally activated by TEF in myeloma cells. Finally, chromatin immmunoprecipitation assays in MM cells confirmed that TEF protein activates directly *Bik* promoter (result not shown). Despite TEF expression, some cell lines do not express Bik, probably due to a potential deletion of *Bik* at 22q13.2, as frequently described in other cancers (Sturm *et al*, 2006). This hypothesis can be further sustained by the frequent chromosomal abnormalities observed in MM (Magrangeas *et al*, 2005). In addition, it was demonstrated that the PAR bZIP pathway mediates oxidative stress-induced apoptosis via Bik regulation. We consequently addressed the role of Bik in H_2_O_2_ oxidative stress response. Indeed, transient Bik expression sensitises myeloma cells to oxidative stress whereas Bik silencing increases resistance to this agent. Another study also demonstrates the implication of Bik in oxidative stress induced by Bz-423 in B cells, however other BH3-only proteins as Bad, Bim and Puma are also implicated in this response (Blatt *et al*, 2009). In addition, we found that ectopic expression of Bik in myeloma cells induces apoptosis. This result is in agreement with previous data, showing that ectopic expression of Bik results in apoptosis of a large number of tumour cells as colon, prostate or melanoma (Gillissen *et al*, 2003; Oppermann *et al*, 2005). Although ectopic expression of Bik seems very effective to induce cell death, its dependence on caspase activation is controversial (Gillissen *et al*, 2003, 2007; Oppermann *et al*, 2005). We found that Bik-induced cell death in myeloma cells is associated with caspase-9 and caspase-3 activation suggesting that Bik promotes cell apoptosis through a mitochondrial–associated caspase pathway in myeloma cells. Moreover, knockdown of Bax or Bak showed that Bik induces cell death via a Bax/Bak-dependent mechanism, indicating that Bax and Bak could have a redundant role in this process. Furthermore, we provide evidence that upon Bik overexpression, Bik is found associated with both Bcl-2 and Bcl-xL and consequently triggers the disruption of Bim/Bcl-2 and Bim/Bcl-xL endogenous complexes. These findings suggest that Bik ectopic expression leads to Bim release that becomes available to exert its pro-apoptotic function through Bax and Bak activation.

Finally, myeloma cells expressing high endogenous Bik/Bcl-2 complexes could exhibit a ‘deadly phenotype’, as previously described for Bim/Bcl-2 pairs (Deng *et al*, 2007). Therefore, small BH3 mimetic molecules should be taken into consideration for further apoptosis-based therapy in myeloma cells that constitutively express endogenous Bik/Bcl-2 complexes.
