# Supplementary material for: BH3-only protein Bik is involved in both apoptosis induction and sensitivity to oxidative stress in multiple myeloma
Source: Br J Cancer. 2010 Nov 9;103(12):1808–14. doi: 10.1038/sj.bjc.6605981 (PMC3008608; doi:10.1038/sj.bjc.6605981)
Supplement: Supplementary Information [file 6605981x1.doc]

Supplemental information

| **HMCL** | **codon** | **nucleotide change** | **exon** |
| --- | --- | --- | --- |
| JIM3 | 273 | CGT>TGT | 8 |
| L363 | insertion |  | Intron 7-8 |
| NCIH929 | wt |  |  |
| U266 | 161 | GCC>ACC | 5 |
| LP-1 | 286 | GAA>AAA | 8 |
| JJN3 | 126 | TAC>AAC | 5 |
| Karpas-620 | 135 | TGC>TAC | 5 |
| KMS-11 | No PCR product |  |  |
| KMS-12PE | 337 | CGC>CTC | 10 |
| KMS-12BM | 337 | CGC>CTC | 10 |
| KMM-1 | 135 | TGC>TGC+TTC | 5 |
| OPM-2 | 175 | CGC>CAC | 5 |
| MM.1S | wt |  |  |
| XG-1 | 126 | TAC>AAC | 5 |
| XG-2 | 176 | TGC>TAC | 5 |
| XG-5 | 282 | CGG>TGG | 8 |
| XG-6 | wt |  |  |
| XG-7 | wt |  |  |
| MDN | wt |  |  |
| NAN-1 | 180 | GAG>TAG | 5 |
| NAN-3 | 248 | CGG>CGG+CAG | 6 |
| NAN-6 | deletion |  | 7-9 |
| SBN | wt |  |  |
| BCN | wt |  |  |

Table 1. *TP53* status of HMCL
